# Population-Genomic Insights into Variation in *Prevotella intermedia* and *Prevotella nigrescens* Isolates and Its Association with Periodontal Disease

**DOI:** 10.3389/fcimb.2017.00409

**Published:** 2017-09-21

**Authors:** Yifei Zhang, Min Zhen, Yalin Zhan, Yeqing Song, Qian Zhang, Jinfeng Wang

**Affiliations:** ^1^Central Laboratory, Peking University School and Hospital of Stomatology Beijing, China; ^2^Department of Periodontology, Peking University School and Hospital of Stomatology Beijing, China; ^3^Computational Genomics Lab, Beijing Institutes of Life Science, Chinese Academy of Sciences Beijing, China

**Keywords:** *Prevotella intermedia*, *Prevotella nigrescens*, periodontitis, comparative genomics, pathogenesis, metabolic function

## Abstract

High-throughput sequencing has helped to reveal the close relationship between *Prevotella* and periodontal disease, but the roles of subspecies diversity and genomic variation within this genus in periodontal diseases still need to be investigated. We performed a comparative genome analysis of 48 *Prevotella intermedia* and *Prevotella nigrescens* isolates that from the same cohort of subjects to identify the main drivers of their pathogenicity and adaptation to different environments. The comparisons were done between two species and between disease and health based on pooled sequences. The results showed that both *P. intermedia* and *P. nigrescens* have highly dynamic genomes and can take up various exogenous factors through horizontal gene transfer. The major differences between disease-derived and health-derived samples of *P. intermedia* and *P. nigrescens* were factors related to genome modification and recombination, indicating that the *Prevotella* isolates from disease sites may be more capable of genomic reconstruction. We also identified genetic elements specific to each sample, and found that disease groups had more unique virulence factors related to capsule and lipopolysaccharide synthesis, secretion systems, proteinases, and toxins, suggesting that strains from disease sites may have more specific virulence, particularly for *P. intermedia*. The differentially represented pathways between samples from disease and health were related to energy metabolism, carbohydrate and lipid metabolism, and amino acid metabolism, consistent with data from the whole subgingival microbiome in periodontal disease and health. Disease-derived samples had gained or lost several metabolic genes compared to healthy-derived samples, which could be linked with the difference in virulence performance between diseased and healthy sample groups. Our findings suggest that *P. intermedia* and *P. nigrescens* may serve as “crucial substances” in subgingival plaque, which may reflect changes in microbial and environmental dynamics in subgingival microbial ecosystems. This provides insight into the potential of *P. intermedia* and *P. nigrescens* as new predictive biomarkers and targets for effective interventions in periodontal disease.

## Introduction

Periodontitis is a term describing specific inflammatory and destructive changes in the tooth-supporting tissues, such as the gingiva, periodontal ligaments, alveolar bone, and root cementum. If not treated appropriately, it can lead to tissue destruction and ultimately tooth loss. Moreover, there is increasing evidence of associations between periodontitis and cardiovascular disease (Stewart and West, 2016), diabetes (Preshaw et al., 2012), and obesity (Moura-Grec et al., 2014). The formation of dental plaque biofilms at the gingival margin is an initiating factor of periodontitis (Socransky and Haffajee, 1994).

The investigation of the microbiology of periodontal diseases spans several decades. Socransky et al. proposed a role for six major microbial complexes in the subgingival biofilm (Socransky et al., 1998) and described the relationship between the addition of species during “microbial succession,” leading to the development of gingival inflammation (Socransky and Haffajee, 2005). Yellow (*Streptococcus* species), green (*Campylobacter concisus, Eikenella corrodens*), purple (*Veillonella parvula*), and blue (*Actinomyces* species) complexes are associated with periodontal health, whereas red (*Porphyromonas gingivalis, Treponema denticola, Tannerella forsythia*) and orange complexes (*Fusobacterium, Prevotella*, and *Campylobacter* species) are closely associated with disease. Although the core members of red complexes (particularly *P. gingivalis*) are considered “keystone pathogens” (Hajishengallis et al., 2012) that can orchestrate periodontitis even at low abundances (Hajishengallis et al., 2011), they need additional changes in the local environment to reach a level to cause disease (Bogren et al., 2007). In a subgingival biofilm redevelopment study, Uzel et al. (2011) found that members of the green and orange complexes, such as *Fusobacterium* subspecies and *Prevotella intermedia*, increased much faster in periodontitis subjects than in periodontally healthy subjects. From days 7 to 21, *F. nucleatum* ssp. polymorphum, *P. intermedia*, and *Prevotella nigrescens* continued to increase, while the levels of members of the red complex did not change significantly even after 21 days. In another study that analyzed 13,261 plaque samples, it was posited that colonization of the subgingival environment by orange complex species was required for the establishment of the red complex species (Socransky et al., 1998). This reminds us that orange complex species may act as instigators to facilitate more pathogenic species of the red complex, and could be targeted as a prevention strategy, resulting in the inhibition of pathogenesis caused by dysbacteriosis.

*P. intermedia* and *P. nigrescens*, member of the orange complex, are among the most frequently encountered species in subgingival plaque (Zambon et al., 1981; Teanpaisan et al., 1995; Kamma et al., 2004). *P. intermedia* is a periodontitis-associated member of the subgingival microbiome, whereas *P. nigrescens* has been detected in most subjects and does not change in proportion from health to disease (Griffen et al., 2012; Abusleme et al., 2013). Thus, it is a core species of the subgingival microbiome (Hong et al., 2015). One study showed that in natural gingivitis, at the start (gingival health) and after 14 days of experimental gingivitis, P. nigrescens is the predominant microorganism (Lie et al., 2001). Gharbia et al. found that *P. intermedia* and *P. nigrescens* may have different site specificities and surface properties and thus their roles in the pathogenesis of periodontitis would be expected to differ (Gharbia et al., 1994).

The pathogenic potential of *P. intermedia* and *P. nigrescens* may vary among strains (Dorn et al., 1998). A *P. nigrescens* strain isolated from a chronic periodontitis lesion produced unique polysaccharides that may play an important role in the development of chronic inflammatory lesions (Yamane et al., 2005). Furthermore, the profile of degradative enzymes produced by the organism may vary depending on its site. Maeda et al. (1998) suggested that *P. intermedia* may increase the activity of degradative enzymes under certain conditions and support the progression of periodontitis.

Recently, much data on the relationship between the oral microbiome and periodontal disease have been generated due to advances in high-throughput sequencing technologies (Duran-Pinedo et al., 2014; Jorth et al., 2014; Li et al., 2014; Kirst et al., 2015), but there are few reports on strain-level detection. Because each bacterial strain in a microbial ecosystem can be considered a genetically defined, basic functional unit (Zhang and Zhao, 2016), focusing on new microbiome findings alone would limit our exploration of their clinical significance. It is necessary to further investigate how each strain in oral ecology, individually or in combination, contributes to the onset and progression of periodontal diseases.

In this study, we hypothesized that *P. intermedia* and *P. nigrescens* may serve as “crucial substances” in subgingival plaque, playing key roles in the local microenvironment. When the matrix is disturbed, the microbial community may become pathogenic. Specific clonal types of strains may be responsible for disease progression, and thus strains that represent relatively avirulent clones may be less effective for maintaining the biofilm. We performed a comparative genome analysis of *P. intermedia* and *P. nigrescens* strains from periodontal disease and healthy sites. We investigated genetic elements specific to each group and their associations with health and disease, and the *Prevotella* pathogenesis mechanisms that might contribute to the onset and progression of periodontal diseases.

## Results

### Clinical strain isolation

**Figure 1:**
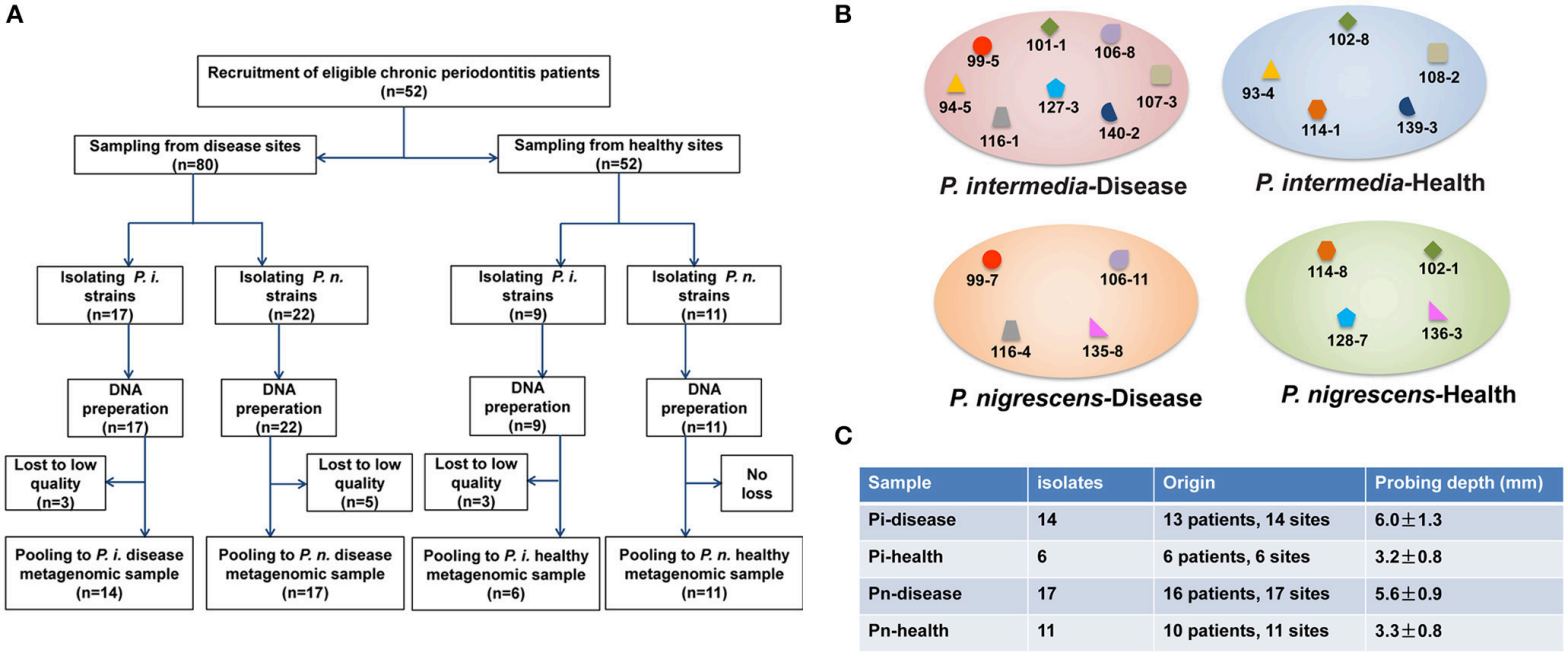
**(A)** Study design overview. **(B)** Clinical isolates from the same patients or even the same sites in each sample group. Isolates with the same label were from the same subject. Isolates with the same former number were from the same sampling site. **(C)** General information on isolates.

An overview of our study design is provided in Figure 1. We analyzed 48 *Prevotella* isolates (Table S1), all taken from different sites from 35 subjects: 14 *P. intermedia* isolates, derived from sites with periodontal disease (Pi-disease group); 6 *P. intermedia* isolates from healthy sites (Pi-health); 17 *P. nigrescens* isolates from diseased sites (Pn-disease); and 11 *P. nigrescens* isolates from healthy sites (Pn-health). In some cases, the same subjects contributed samples of healthy and diseased areas.

### General genome features of pooled samples

Genome sequencing data were generated from four pooled samples, as described below. The general genome features of *P. intermedia* and *P. nigrescens* samples from diseased and healthy sites are listed in Table 1. About 80% of reads of clinical samples of *P. intermedia* could be mapped to the *P. intermedia*-17 (GenBank number: NC_017860.1/NC_017861.1) reference genome, whereas the remainder had best matches in various species, such as other *Prevotella* species, *Bacteroides* species, *P. gingivalis, T. forsythia, Capnocytophaga* species, *Eubacterium* species, *Flavobacterium* species, *Alistipes* species, and *Fusobacterium* species. Major differences between Pi-disease and Pi-health sample groups were that partial reads in Pi-disease can be mapped to *Acidithiobacillus ferrivorans, Anaerofustis stercorihominis, Mycoplasma fermentans, Ruminococcaceae* species and *Treponema* sp., while some reads in Pi-health mapped to *Enterobacter cloacae* and *Xanthomonas fragariae*.

**Table 1:** Summary of analyzed genome data.

**Sample**	**Total reads**	**Mapped reads**	**Total bases**	**Mapped bases of reference**	**Coverage (%)**
Pi-disease	40,287,600	33,417,719	3,824,344,295	2,554,975	94.65
Pi-health	23,341,144	18,767,547	2,160,552,995	2,528,402	93.66
Pn-disease	23,735,598	19,687,748	2,293,047,725	2,586,054	96.89
Pn-health	27,985,784	23,417,711	2,705,008,273	2,550,345	95.55

*The reads of P. intermedia samples were mapped against the reference P. intermedia 17. The reads of P. nigrescens samples were mapped against reference P. nigrescens ATCC 33563*.

Regarding the *P. nigrescens* samples, ~20% of the reads mapped to species other than the reference strain *P. nigrescens* ATCC33563 (GenBank number: NZ_AFPX00000000.1), including *Prevotella* species, *Bacteroides* species, *P. gingivalis, T. forsythia, Alistipes* species, *Odoribacter splanchnicus, Neisseria* species, *Parabacteroides distasonis, Flavobacterium* species, *Tannerella* species, *Treponema* species, *Streptococcus agalactiae*, and *Faecalibacterium prausnitzii*. Major differences between Pn-disease and Pn-health samples were that some reads in the Pn-disease group can be mapped to *Bergeyella zoohelcum, Chryseobacterium* species, *Clostridiales* species, while some reads in healthy group mapped to *Acinetobacter baumannii* and *Saccharomyces cerevisiae*.

**Figure 2:**
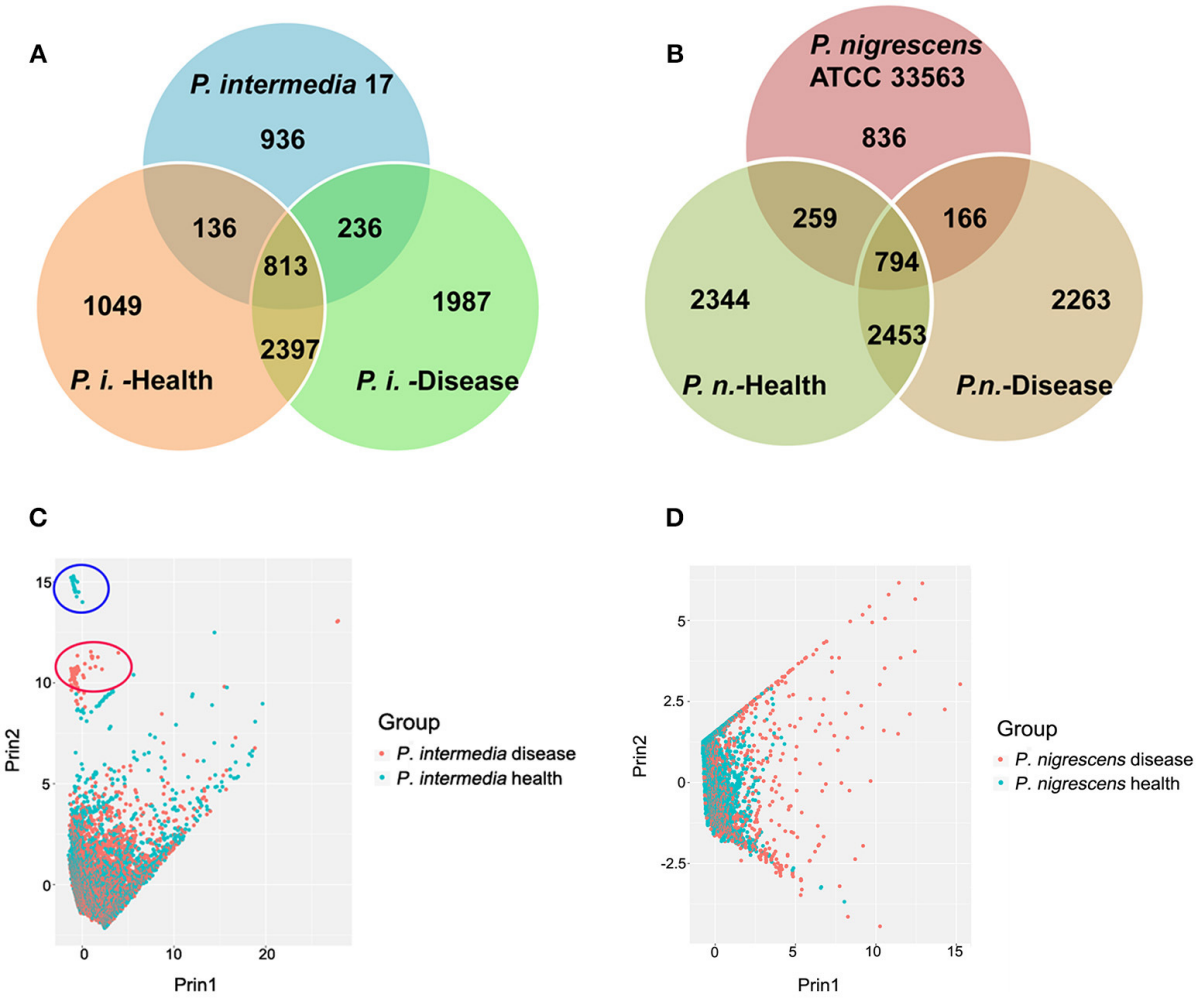
**(A,B)** Orthologous gene contents of **(A)** reference sequence (*P. intermedia*-17), Pi-disease, and Pi-health. **(B)** reference sequence (*P. nigrescens* ATCC 33563), Pn-disease, and Pn-health. **(C,D)** Inter-sample sequences diversity of **(C)**. Pi-disease and Pi-health. **(D)**. Pn-disease and Pn-health.

Open reading frames (ORFs) likely to encode proteins were predicted according to NCBI Prokaryotic Genomes Annotation Pipeline (Tatusova et al., 2016). Then all predicted proteins were analyzed for sequence similarities against the Uniprot database (The UniProt Consortium, 2015). The total predicted ORFs in each group were: Pi-disease: 8020; Pi-health: 6255; Pn-disease: 6943; and Pn-health: 7710. There were 813 orthologous genes shared by the Pi-disease, Pi-health, and reference sequences (Figure 2A). In total, 3210 genes were shared between the Pi-disease and Pi-health groups, whereas Pi-disease samples had more group-specific genes than Pi-health samples (1987 vs. 1049). Totally 794 orthologous genes were shared by the Pn-disease, Pn-health, and reference sequences. Both diseased and healthy groups of *P. nigrescens* had almost the same numbers of group-specific genes (2344 vs. 2263), and shared 3247 genes between Pn-disease and Pn-health groups (Figure 2B).

Based on the lengths of matched segments in sample sequences, the percent identity of matched segments to reference, the full length of the matched contig, the degree of coverage of matched segments to reference, and the degree of coverage of matched segments to contigs, we investigated inter-sample diversity by performing a principal component analysis (PCA) of Pi sample groups (Figure 2C) and Pn sample groups (Figure 2D). Between Pi-disease and Pi-health samples, most contigs could not be differentiated (Figure 2C), indicating that overall there were no obvious differences in genome sequences between the groups that matched the reference. However, two additional small clusters of samples were clearly distinct (Figure 2C), consisting of contigs from each group. The contigs in the two distinct clusters were identified in Table S2, which were all belonged to the predicted insert sequences of Pi-disease or Pi-health samples. We also identified the annotated genes in these distinct contigs, most of which were conjugal transfer proteins (e.g., TraG, TraA, TraB, TraD etc. for Pi-disease group; TraE, TraK, TraM, and TraN for Pi-health group), phage associated proteins (both groups), recombinase (both groups), peptidase (Pi-health group), DNA-binding protein (Pi-disease group), and tetracycline resistance genes (e.g., tet Q and tet R; for Pi-disease group). Regarding the Pn group, most contigs from Pn-health samples clustered with those from Pn-disease; however, a considerable proportion of contigs in the Pn-disease group were clearly distinct from those in the Pn-health group (Figure 2D).

### Unique genes in each group

**Figure 3:**
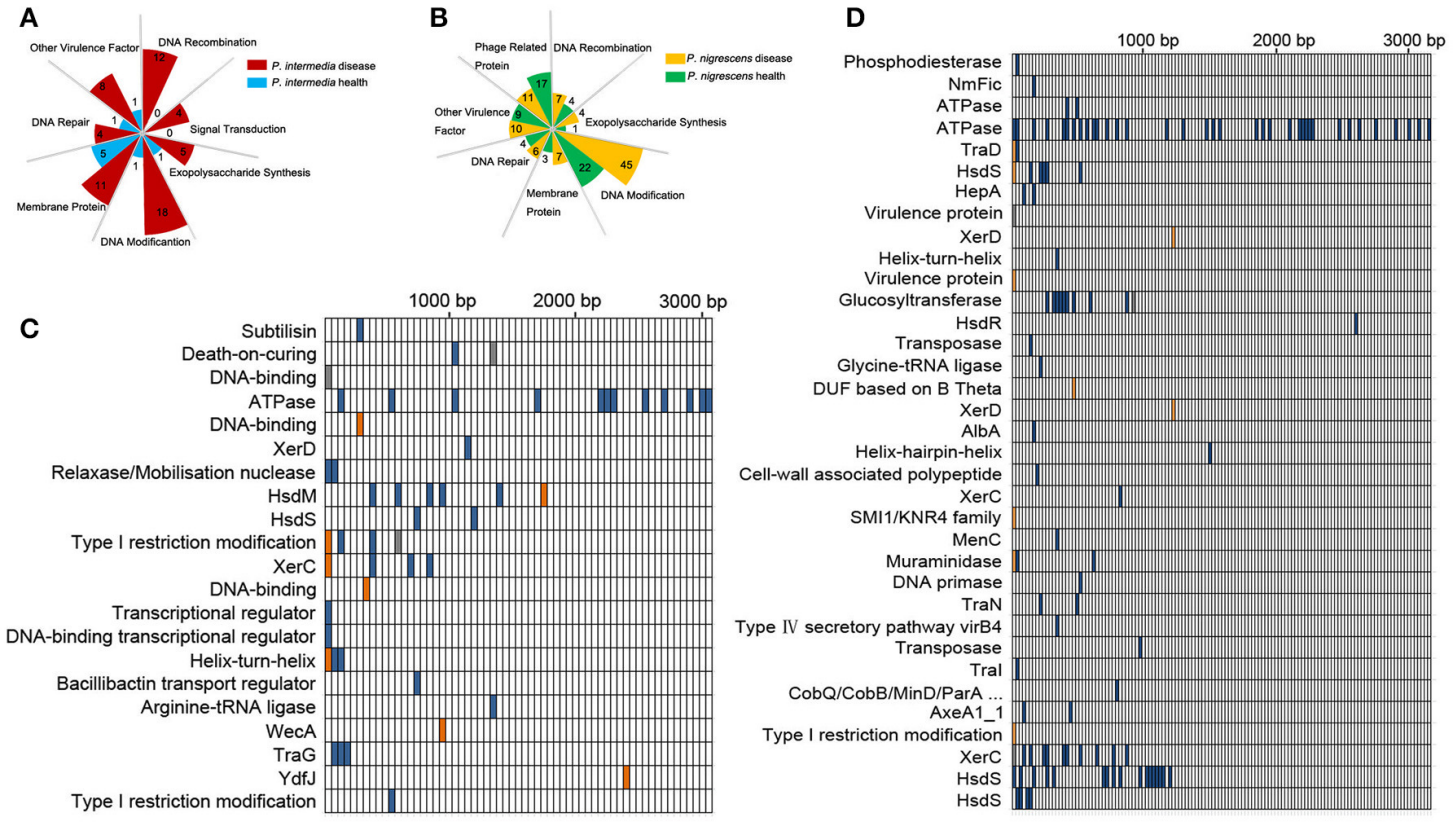
**(A,B)** Functional categories of unique genes in Pi-disease/Pi-health groups **(A)** and Pn-disease/Pn-health groups **(B)**. The number in each square indicates the counts of genes in each category. **(C,D)** High-effect SNV distribution in each CDS in Pi-health sample (vs. Pi-disease sample) **(C)** and Pn-health sample (vs. Pn-disease sample) **(D)**.

Unique Pi genes are listed in Tables S3, S4. The Pi-disease group had more unique genes involved in DNA modification, DNA recombination, DNA repair, and signal transduction, and more virulence-related factors (Figure 3A). Moreover, there were differences in exopolysaccharide biosynthesis and membrane proteins between the Pi-disease and Pi-health groups. Unique Pn genes are listed in Tables S5, S6. The dominant genes differentiating the groups were related to DNA modification, phage proteins, DNA recombination, and DNA repair (Figure 3B). The Pn-disease group had many more specific genes that functioned in DNA modification, and more specific phage-related proteins were found in the Pn-health samples, but both groups had almost the same number of unique genes related to virulence. There were also differences in exopolysaccharide biosynthesis and membrane proteins between the groups.

### Single-nucleotide variants in core regions

To investigate functional differences in orthologous genes between the Pi groups, we detected single-nucleotide variants (SNVs) in coding sequences (CDSs), and screened those whose functions were predicted to be highly affected (Figure 3C). The most affected regions were those encoding CDSs involved in DNA binding, DNA modification, and recombination, including HsdM, HsdS, XerC, XerD, and Type I restriction modification DNA-specific domain protein. The dominant regions that were affected by SNVs in the Pn-health (vs. Pn-disease) group were those encoding CDSs involved in ATPase, DNA modification (HsdS, HsdR), DNA recombination (XerC, XerD), conjugate transposon (TraD, TraN, TraI), and virulence protein (Figure 3D).

### Label sequences in each group

We also identified unique sequences in each sample, and tried to find any discrepant virulence gene candidates using BLASTX searches against the virulence factor database (VFDB; last accessed Nov 25, 2016; Chen et al., 2012) under a threshold e-value (≤ 1e-5). The numbers of unique sequences and matched virulence factors in each group are listed in Table S7. Most virulence factors specific to disease groups were capsule and lipopolysaccharide (LPS) synthesis-related factors. Moreover, disease groups had more unique sequences related to secretion systems, where they and the diverse proteins they secreted represent critical determinants of competitive fitness and pathogenic potential. The most abundant specific gene cluster in the Pi-disease group was the Type VI secretion system (T6SS). Pn-disease samples had specific sequences mapped to T3SS. Both the Pn-disease and Pn-healthy groups had T4SS effectors, but with different sequences. BspA and HlyB existed in all groups (health as well as disease), but with unique sequences in each sample group.

Furthermore, disease groups had more specific virulence factors annotated as proteinases and toxins. Only Pi-disease and Pn-disease samples possessed specific sequences that could match proteasome-associated ATPase (mpa gene).

### Metabolic pathway reconstructions

**Figure 4:**
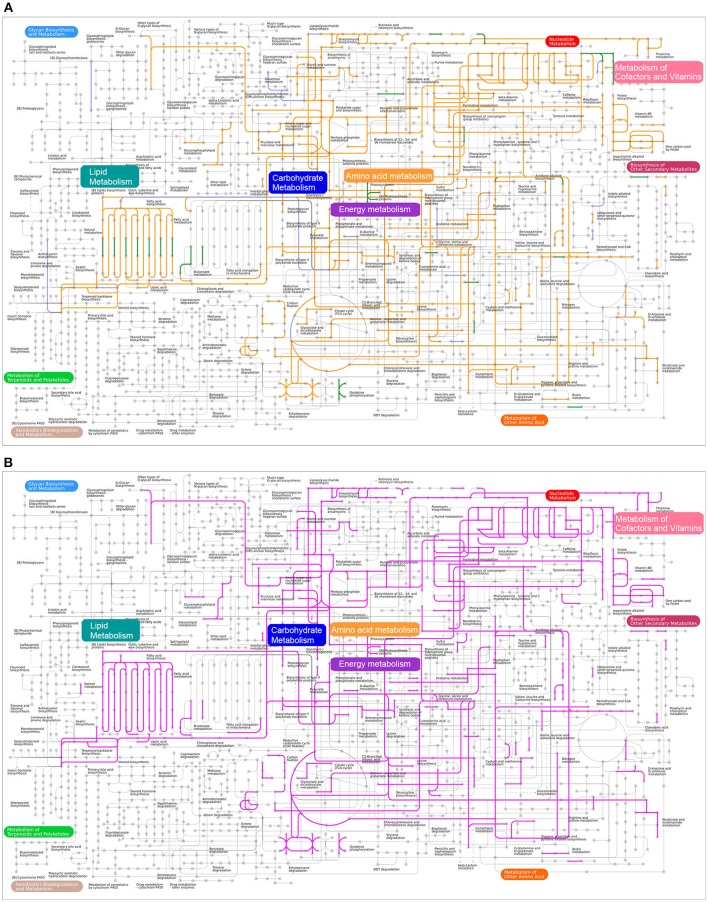
**(A)** Metabolic pathway reconstructions of Pi-disease and Pn-disease. Pipelines colored in orange represent the common pathways shared by both samples. The pipelines colored in purple and green represent the specific pathway in Pi-disease and Pn-disease, respectively; **(B)** Metabolic pathway reconstructions of Pi-health and Pn-health. Pipelines colored in pink represent the common pathways shared by both samples; no specific pathway was seen in Pi-health or Pn-health.

**Figure 5:**
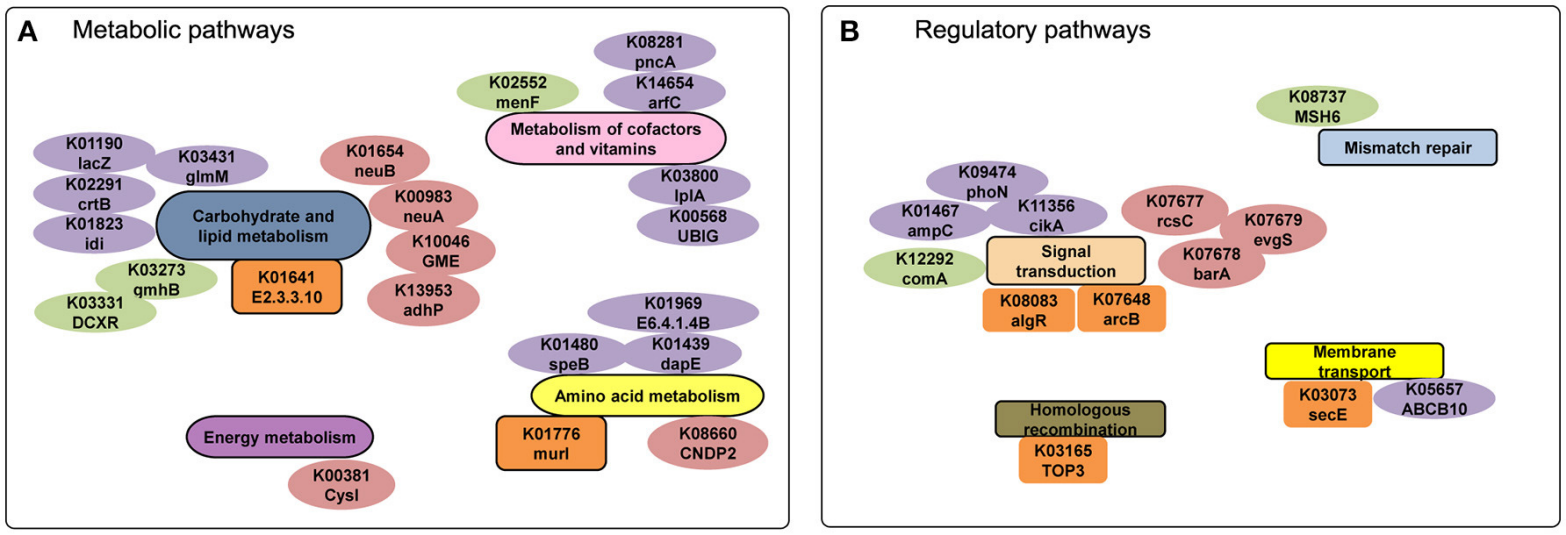
**(A)** Metabolic and **(B)** regulatory analyses of diseased-derived and healthy-derived samples. The gene labels in the small pathway groups are orange (only exist in disease samples) or pink (only exist in healthy samples). The labels colored in purple and green represent the specific genes only exist in Pi-disease and Pn-disease.

We performed a comparison of metabolic networks between combined disease (Pi, Pn- disease) and combined health samples (Pi, Pn- health). We first determined the functional gene orthologs (KOs) in metabolic and regulatory pathways that shared by Pi-disease and Pn-disease (disease-share), and by Pi-health and Pn-health (health-share). Then we identified the differential KOs involved in networks between disease-share and health-share. We also managed to define special metabolic and regulatory characteristics of each sample group. (Figures 4, 5). The metabolic pathway of Pi-disease is highly consistent with that of Pn-disease, except for a few differences that mainly involved in energy metabolism, carbohydrate metabolism, and amino acid metabolism. In regulatory pathways, the major differences were seen in signal transduction (Figures 4A, 5, Figure S1) Pi-health and Pn-health shared all the KOs so they had exactly the same metabolic and regulatory pathways (Figure 4B, Figure S1B). Disease-share had specific KOs involved in the generation of 3-hydroxy-3-methglutaryl-CoA through valine, leucine and isoleucine degradation (e.g., E.2.3.3.10), and amino acid metabolism (e.g., murI), while health-share had specific KOs involved in pathways including Sulfur metabolism (e.g., cysI), glycolysis/gluconeogenesis (e.g., adhP), amino acid metabolism (e.g., CNDP2), amino sugar and nucleotide sugar metabolism (e.g., neuA, neuB, GME) (Figures 4, 5A). In addition, there was a distinct difference in signal transduction between disease-share and health-share. Both had unique two-component systems: e.g., algR and arcB in diseased samples and evgS, rcsC, and barA in healthy samples (Figure 5B, Figure S1).

We identified the KOs that merely exist/absent in Pi-disease or Pn-disease samples as well. Compared to other three sample groups, Pi-disease samples had gained specific genes involved in carbohydrate and lipid metabolism (e.g., lacZ, crtB, idi, and glmM), metabolism of cofactors and vitamin (e.g., arfC, UBIG, pncA, and lplA) and amino acid metabolism (e.g., speB, dapE, and E6.4.1.4B) (Figures 4, 5), while Pn-disease samples had extra genes with specific pathways including lipopolysaccharide biosynthesis (e.g., gmhB), pentose and glucuronate intercoversions (e.g., DCXR), and ubiquinone biosynthesis (e.g., menF). In regulatory pathways, Pi-disease samples had more unique genes involved in two-component system (e.g., phoN, ampC, and cikA) and membrane transport (e.g., ABCB10). Pn-disease had more unique genes involved in mismatch repair (e.g., MSH6) and two-component system (comA) (Figure 5). On the other hand, Pi-disease samples have also lost several genes, which involved in metabolism of cofactors and vitamin (e.g., K00077 apbA, K01719 hemD, K01495 folE, and K00652 bioF), fatty acid metabolism (e.g., K02372 fabZ, and K09478 ACADSB), and nucleotide metabolism (e.g., K01588 purE and K01493 comEB). There was only one gene ortholog that absent in Pn-disease samples only, which is involved in lipopolysaccharide metabolism (K02535 LPXC).

### Function of unique gene clusters

**Figure 6:**
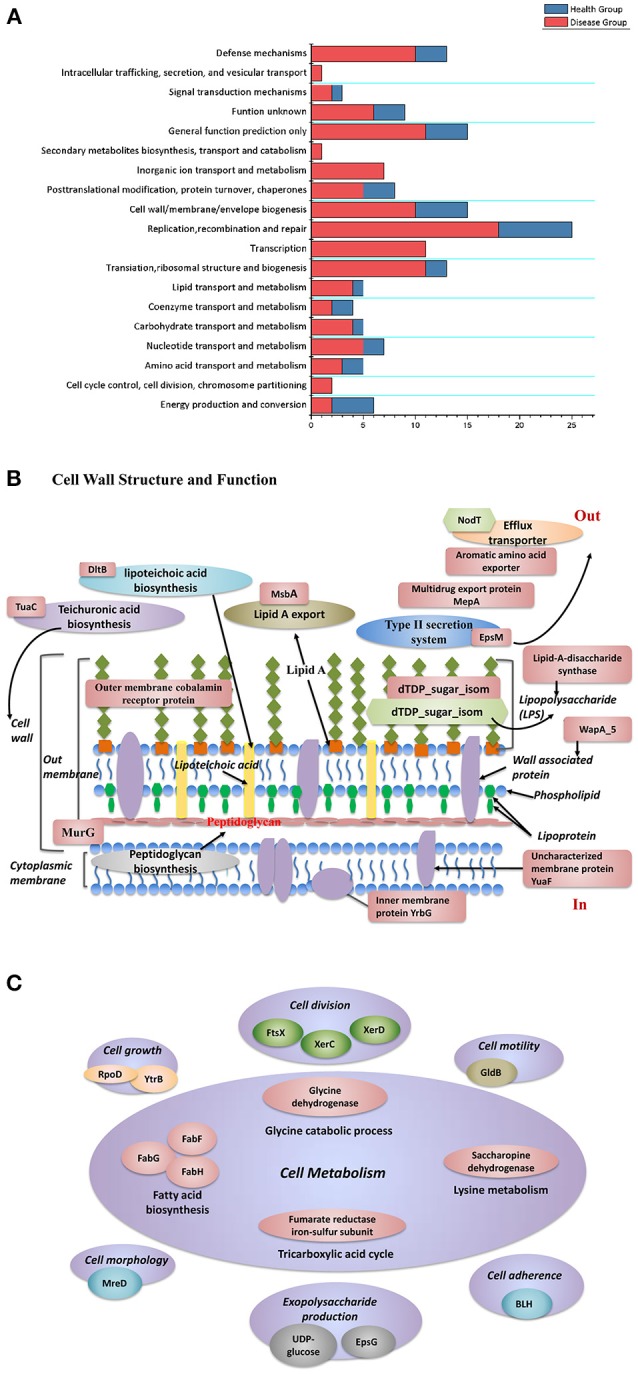
**(A)** Functional classification of unique gene clusters between diseased and healthy groups. **(B)** The distribution of protein functions that differed between disease-derived and healthy-derived isolates. Red labels represent factors unique in disease-derived isolates, and green labels represent factors unique in healthy-derived isolates. **(C)** Proteins that function in cell metabolism that were unique in the disease group.

To understand the functions of different genes among the disease-derived (combined Pi,Pn- disease) and healthy-derived (combined Pi, Pn- health) isolates, protein functions were examined for clusters that were unique to isolates from diseased and healthy sites, based on the clusters of orthologous groups (COG) database (Tatusov et al., 2001) (Figure 6A). Diseased samples were enriched in 17 COGs, mostly related to gene regulation, signal transduction, biosynthesis, and function of cell wall structure, cell growth processes, and transport or metabolism functions, whereas healthy samples were enriched in only 1 COG related to energy production and conversion. The specific genes are listed in Tables S8, S9 and Figure 6. Diseased isolates had more unique factors related to cell wall and outer membrane structure and function, such as teichuronic acid biosynthesis glycosyltransferase (TuaC), D-alanyl-lipoteichoic acid biosynthesis protein (DltB), and the outer membrane cobalamin receptor protein. Others included the lipid A export ATP-binding/permease protein (MsbA), acetyltransferase (EpsM), lipid-A-disaccharide synthase, and dTDP-4-dehydrorhamnose 3,5-epimerase (dTDP_sugar_isom), which are involved in the pathway of LPS biosynthesis (Figure 6B).

Regarding microbial growth, disease-derived samples had more unique factors that influence cell division, cell metabolism, cell morphology, cell motility, and exopolysaccharide biosynthesis (Figure 6C). Diseased samples of both species contained an unusual YpdA/YpdB two-component regulatory system involved in a carbon control network whereas healthy isolates did not. In particular, isolates from diseased groups had a unique anaerobic ribonucleoside-triphosphate reductase activating protein, which is activated under anaerobic conditions, whereas healthy groups had a unique fumarate hydratase (class I, aerobic) that functions as an aerobic enzyme, involved in the citric acid cycle. Moreover, isolates from both diseased and healthy sites possessed different DNA methylases (Tables S8, S9). Diseased samples had more unique gene clusters that could aid resistance to stress factors, such as arsenate reductase and arsenic-resistance protein, which can enhance resistance to arsenate; heat-shock protein, which may help cells recover from stress; Na^+^/drug antiporter; and cold-shock DEAD box protein. Isolates from both types of isolates had different FeS cluster assembly proteins including SufB, which helps facilitate iron uptake from extracellular iron chelators under iron limitation.

LPSs are important factors involved in the virulence of *Prevotella* species, and we found that both diseased and healthy samples had several different factors directly or indirectly related to LPSs. Thus, we carried out a comprehensive analysis of LPS biosynthesis proteins in type culture strains from the NCBI database and clinical strains from each group of this study by reconstructing a neighbor-joining phylogenetic tree with MEGA 6 (Tamura et al., 2013) (Figure S2). LPS biosynthesis proteins from *P. intermedia* and *P. nigrescens* isolates clustered together. Notably, four disease-derived isolates clustered within a highly distinct and uniform clade.

### Mobile genetic elements

Each group had many mobile genetic elements. We counted the number of conjugate transposons (CTns), transposases (Tns), and integrases, and divided by the number of isolates contained in each group to calculate the mobile genetic elements (MGE)/isolates ratio (Table 2). The ratio of CTns to isolates in Pi-health was higher than that in Pi-disease group, while the ratio in Pn-disease and Pn-health groups were similar. The ratios of Tns and integrase to isolates were higher in healthy groups (both in Pi-health and Pn-health) than that in disease groups. We also found that each group contained several prophages or degraded prophages, with Pn-disease and Pi-health samples having more than Pi-disease and Pn-health samples.

**Table 2:** Mobile genetic elements in each group.

	**Pi-disease**	**Pi-health**	**Pn-disease**	**Pn-health**
^*^CTns/isolates	41/14 = 2.93	22/6 = 3.67	44/17 = 2.59	28/11 = 2.55
Tns/isolates	60/14 = 4.29	34/6 = 5.67	61/17 = 3.59	66/11 = 6
Integrase/isolates	40/14 = 2.86	24/6 = 4	56/17 = 3.29	52/11 = 4.73
^#^Prophages	4	3	10	2

* *CTns were calculated by the number of protein that found in conjugate transposon*.

# *Prophages included intact prophages and defective prophages*.

### CRISPR array sequences

The numbers of spacers in Pi-disease, Pi-healthy, Pn-disease, and Pn-healthy samples were 577, 183, 980, and 522, respectively. In Pi-disease and Pn-disease samples, 4.7 and 4.8% spacers showed significant nucleotide similarity to the phage database, slightly higher than for Pn-healthy (4.4%) and Pi-healthy (2.7%) samples. More spacers matched the plasmid database in Pi-disease (3.3%) and Pi-healthy (3.3%) samples than in Pn-disease (2.7%) and Pn-healthy (2.9%) samples (Figure S3).

## Discussion

The oral cavity is one of the most biologically complex sites in the body, having a natural microflora with a characteristic composition (Kurtz et al., 2004; Nasidze et al., 2009). Normally, the oral microflora exists harmoniously with the host, and will only cause disease (most commonly, caries and periodontitis) when homeostasis breaks down (Socransky and Haffajee, 2005). Clarifying the exact mechanism(s) by which the commensal microorganisms become pathogenic could open new opportunities for more effective disease prevention, treatment, and control. Most studies that have investigated the issue have focused on the diversity in the oral microbial ecology, making comparisons between patients and healthy populations (Human Microbiome Project Consortium, 2012; Yang et al., 2012; Wang et al., 2013). However, because host susceptibility is a key factor in oral disease, the impact of population variation on such results should not be ignored. Thus, in our study, we collected isolates from different sites from patients in the same population (those with periodontitis) to minimize this issue.

Full-genome sequencing of individual strain can provide more complete information indeed, but this approach is impractical for large population samples. It may be unable to understand how the genomes of these different strains are similar to or differ from each other through “pooled-sequencing,” however, our original intention was to consider the isolates that from disease or health as a whole and determined the unique functional genes (e.g., involved in metabolic pathway and virulence) that merely exist/absent in diseased or healthy sites at population level. And “pooled-sequencing” would have little impact on the presence or absence of certain genes in each group because of the high sequencing coverage. Besides, those candidate genes could be further verified in more individual isolates. Since *P. intermedia* distributes more prevalent in diseased sites than in healthy sites, thus we obtained much less healthy-derived *P. intermedia* isolates than diseased-derived. However, our sequencing depth was higher than 800X, so all the isolates in each group would be covered. The unique genes or orthologs comparison was relied on the presence or absence, not on the quantity. Thus, we did not consider the difference in gene or orthologs abundance.

The genome of *P. intermedia* is unusually enriched in CTns and MTns (Belda-Ferre et al., 2012), suggesting a high level of genomic plasticity. In this study, all samples showed many mobile genetic elements, exogenous insert sequences, and proteins related to prophages, indicating a high occurrence of transfer events (e.g., conjugation, transduction, and transformation). This may be one explanation why the genomes of *P. intermedia* and *P. nigrescens* were not conserved and showed high genomic diversity. The environment under periodontal disease conditions is more diverse than that under healthy conditions (Naito et al., 2016). However, in this study, the horizontal transfer events seemed more likely to occur under healthy conditions. The matches of spacers to phage and plasmid sequences suggested that higher ratio of phage/plasmid space has more frequent encounter of exogenous extracellular DNA. Since each unique spacer in CRISPR is acquired from invasive foreign genetic elements, such as phages and plasmids, our data indicated that the host genomes of *P. intermedia* and *P. nigrescens* have acquired immunity against such phages or plasmids, and also serve as a record of their interactions with phages and plasmids. Under diseased conditions, bacterial hosts may interact more often with phages, and thus gain enhanced resistance to phage attack. However, the interaction between host and plasmid may depend on species.

We also found major differences in factors related to genome modification and recombination between disease-derived and health-derived samples in each species, indicating that the *Prevotella* isolates from disease sites may be more capable of genomic reconstruction. We also determined the unique sequences in each group. Our results indicate that *P. intermedia* and *P. nigrescens* in diseased samples had more specific virulence factors related to the cell capsule and LPS. Signal transduction was another key difference between diseased and healthy samples. The type VI secretion system (T6SS) shows intriguing versatility, targeting effector proteins to both eukaryotic cells and competitor bacterial cells (Shi et al., 2015). Many pathogens use it as a weapon to compete against rival bacterial cells by injecting them with multiple antibacterial toxins (Ho et al., 2014). In our study, only the Pi-disease group had unique sequences that matched T6SS-related proteins, indicating that Pi-disease samples may have specific pathogenic properties based on T6SS. The Type III secretion system (T3SS) has two homologous families: the flagellar T3SS drives cell motility, and the non-flagellar T3SS is a sophisticated molecular machinery of bacteria used to inject (translocate) bacterial proteins (effectors) into eukaryotic cells, a trait frequently associated with virulence (MacIntyre et al., 2010). Thus, Pn-disease samples may express unique virulence through T3SS. The effectors of secretion systems play important roles in the pathogenesis of bacteria, and different effectors may have different functions (Enninga and Rosenshine, 2009). We found that both Pn-disease and Pn-healthy samples had T4SS effectors, but with different sequences. Whether this reflects a distinction in virulence between the two sample types may be worthy of further investigation.

Bacteroides surface protein A (BspA) is a secreted surface protein belonging to the leucine-rich repeat (LRR) family (Siamer and Dehio, 2015). It is a well-defined virulence factor of *T. forsythia* (Sharma et al., 1998) that may trigger the release of bone-resorbing pro-inflammatory cytokines from monocytes and chemokine IL-8 from gingival epithelial cells. It has also been shown to mediate bacterial adherence and invasion into epithelial cells. HlyB is thought to be closely related to multi-drug resistance. We found that the disease groups had more specific virulence factors annotated as proteinases and toxins, among which BspA and HlyB were common in all groups but with different sequences. Another virulence factor that should be highlighted is proteasome-associated ATPase (mpa gene). This protein is involved in the proteasomal Pup-dependent pathway, which is involved in protein degradation and pathogenesis (Darwin et al., 2005; Sharma, 2010). In our study, only the disease groups had specific sequences matching mpa.

Previous studies didn't show any difference in amino acid metabolism and glucose metabolism between *P. intermedia* and *P. nigrescens* (Takahashi and Yamada, 2000a,b). And our results confirmed that *P. intermedia* and *P. nigrescens* strains derived from healthy sites had common metabolic and regulatory networks from the overall aspect. However, disease-derived samples had gained or lost several metabolic genes compared to healthy-derived samples. It is reported that pathogens always require new metabolic pathways that allow them to exploit available food sources in order to thrive in new environment (Schmidt and Hensel, 2004), and the loss of non-essential metabolic functions could contribute to virulence by putting less demand on metabolic pathways (Rohmer et al., 2011). Thus, the metabolic function could be linked with the difference in virulence performance between diseased and healthy groups, and pathways could potentially be good targets for anti-microbial therapies (Rohmer et al., 2011). Besides, our results showed that Pi-disease samples gained/lost more genes than Pn-disease samples, indicating *P. intermedia* could be more adaptable to disease environment. Recent surveys on the subgingival microbiome in periodontitis suggest that the differentially represented pathways between periodontal disease and health are related to energy metabolism (e.g., aminoacyl-tRNA biosynthesis, nitrogen metabolism), carbohydrate and lipid metabolism (e.g., glycolysis/gluconeogenesis, citrate cycle, pyruvate metabolism) (Duran-Pinedo et al., 2014), and purine and pyrimidine metabolism (Kirst et al., 2015). Our data are consistent with those results regarding metabolic differences between disease and healthy samples.

The major differences in GO categories between the diseased and healthy groups were in transporters and DNA repair and recombination proteins, also consistent with a recent study (Kirst et al., 2015). Moreover, both diseased groups had several specific factors that have been reported to be more highly expressed in periodontitis subjects, such as Btu, Clp, Fts, RpoD, and homologs to internalins (Duran-Pinedo et al., 2014). Disease-derived isolates had more unique gene clusters whose functions were related to drug and metal resistance (e.g., zinc metalloprotease, ABC transporter), also consistent with a previous report (Duran-Pinedo et al., 2014). The ability of *P. intermedia* and *P. nigrescens* to alter their metabolic characteristics depending on the nutrients available has been confirmed (Krakoff, 2006). These relationships could indicate that the functional potential of the subgingival microbiome under different health conditions might be determined according to the *P. intermedia* and *P. nigrescens* in the subgingival plaque.

The high level of plasticity and diversity of the *Prevotella* genomes has been demonstrated in other studies (Saito et al., 2001; Purushe et al., 2010). However, different strains may be affected by different exogenous factors, which could facilitate adaptation to varied environments and available nutrients, and generate virulence. This may be why *Prevotella* is associated with various infectious diseases, such as endodontic infections (Ruan et al., 2015), acute apical abscesses (Hsiao et al., 2012), apical periodontitis, dental implant failure (Siqueira and Rocas, 2013), and even cystic fibrosis (Dingsdag et al., 2016). In our study, the species-level comparative analysis of *Prevotella* species suggests that members of the “orange complex” may be able to capture important functional differences across samples and those differences could reflect changes in microbial and environmental dynamics in subgingival microbial ecosystems. We should consider *P. intermedia* and *P. nigrescens* as new biomarkers with predictive value, as well as new targets for effective interventions in periodontal disease.

## Methods

### Sample collection and isolation of *P. intermedia* and *P. nigrescens*

Patients with chronic periodontitis were recruited from the Department of Periodontology, Peking University School and Hospital of Stomatology. Enrollment occurred between December 2013 and May 2015. The inclusion criteria were: good general health and not pregnant, aged 18 to 65 (male or female), had not taken any antibiotic or antimycotic compounds in the past 3 months or undergone any periodontal therapy within 1 year, no aggressive form of periodontal disease, and periodontal disease sites at least 5 mm probing depth and attachment loss >0 mm. Subjects were excluded if they were pregnant, had any systemic condition which could affect the progress of periodontal disease. Periodontally healthy sites require a probing depth not >4 mm and no attachment loss.

The study protocol was approved by the institutional review board of Peking University School and Hospital of Stomatology (Beijing, China) (approval number: PKUSSIRB-2012063).

For each subject, subgingival plaque samples were collected from gingival pockets with a sterile Gracey curette from the bottom of the pocket to the coronal portion of the pocket. Samples were placed in individual tubes containing 500 μL Ringer's solution and then inoculated onto trypticase soy agar supplemented with 5% sheep blood, 1 mg/mL yeast extract, 1 μg/mL menadione, and 5 μg/mL hemin. Plates were grown anaerobically (90% N2, 5% H2, 5% CO2) at 37°C for 5 days, and black-pigment clones were selected and subcultured for purity. *P. intermedia* and *P. nigrescens* isolates were identified through full-length 16S rDNA sequencing (Sangon Biotech, Shanghai, China). Briefly, the full-length 16S rDNA region was amplified with a universal primer set (27forward, 5′-AGAGTTTGATCCTGGCTCAG-3′; 1492reverse, 5′-GGTTACCTTGTTACGACTT-3′). The PCR condition is as follows: 5 min initial denaturation at 95°C; 35 cycles of denaturation at 95°C (30 s), annealing at 60°C (30 s), elongation at 72°C (90 s); and final extension at 72°C for 10 min. The resulting 16SrRNA sequences were searched against the National Center of Biotechnology Information (NCBI) database, and assigned to species based on the best match at greater than 97% nucleotide identity over at least 95% length of the query.

### Genome sequencing and annotation

Genomic DNA of each isolate was extracted using the Qiagen DNA Purification Kit following the protocol. After checking quality, genomic DNA was pooled according to the study design, and then a sequencing library was built and sequenced on an Illumina HiSeq 2500 system (Biotechnology Corporation, Shanghai, China). Approximately 23–40 million paired-end reads (2 × 125 nt multiplex) were obtained, representing >100-fold genome coverage. Genome mapping to reference genomes was performed using the Burrows-Wheeler Aligner software (ver. 0.7.12). Contigs were assembled de novo using SPAdes-3.5.0 (Bankevich et al., 2012), and filtered by deleting contigs with coverage ≤ 10% and length < 500 bp. Then we performed a cluster analysis of remained contigs using CD-HIT version 4.6 (Fu et al., 2012). Contigs with similarity ≥90% were compared, and only the longest one reserved. To understand the relationship between the contigs and the reference genome, we aligned the contigs to the reference genomes (*P. intermedia* samples to *P. intermedia* 17 and *P. nigrescens* samples to *P. nigrescens* ATCC 33563), and the lengths of matched segments in sample sequences, the percent identity of matched segments to reference, the full length of the matched contig, the degree of coverage of matched segments to reference, and the degree of coverage of matched segments to contigs were recorded for principal component analysis.

The draft genome was annotated using the NCBI Prokaryotic Genomes Annotation Pipeline (Tatusova et al., 2016). The remaining un-mapped reads of four samples were searched by BLASTN program against NCBI non-redundant nucleotide database with an E-value threshold of 1E-5.

### Unique genes prediction

To determine the unique genes in each sample group, we first performed alignments using the pan-genomes analysis pipeline (PGAP) among those four sample groups. The chosen standard was GF, score: 40, identity: 0.6, and coverage: 0.8. We further carried out a group-to-group BLASTN search. For example, the genes obtained from Pi-disease sample group were aligned with the genome sequences in Pi-health sample by BLASTN search, and the results were further filtered by deleting genes with identity > 30% and coverage > 30%, then we obtained a unique genes list for Pi-disease sample group.

### Label sequences identifying in each group

To identify unique sequences in each sample, the sequences in each sample group were compared with all the other sample groups through MUMmer 3.23(Kurtz et al., 2004) (for example, Pi-disease vs. Pi-health; Pi-disease vs. Pn-disease; and Pi-disease vs. Pn-health). The sequences (≥500 bp) that existed in Pi-disease but absent in any other groups were considered as label sequences of Pi-disease group. Label sequences in each group were further annotated by BLASTX searching against the virulence factors database (VFDB, last accessed: November 25, 2016; Chen et al., 2012) with a word size of 10 under the thresholds of the highest bit score and e-value (≤ 1e-05).

### Clustered regularly interspaced short palindromic repeat analysis

Clustered regularly interspaced short palindromic repeat (CRISPR) regions were identified from contigs using the CRISPRs Finder online (Grissa et al., 2007). The sequences of CRISPR direct repeats and spacers in the four sequenced samples were extracted. To characterize a target of each spacer sequence, the spacer list was subjected to a BLASTN search with a word size of seven against the plasmid and the viral database, as described previously (Pride et al., 2012; Rho et al., 2012; Watanabe et al., 2013; Zhou et al., 2015; Wang et al., 2016). Hits were considered significant under the threshold of the e-value (≤ 1e-02).

### Metabolic reconstruction

The metabolic and regulatory pathways were reconstructed using 146 KEGG pathways and 22 KEGG regulatory pathways. An overview of the complete metabolism in biological systems was provided through the web-based interactive tool “Pathways Explorer” (iPath) (Yamada et al., 2011).

### Mobile genetic elements prediction

The assembled contigs were compared to reference genomes (*P. intermedia* 17 and *P. nigrescens* ATCC 33563) through MUMmer 3.23 (Kurtz et al., 2004), and sequences under the thresholds of length ≥500 bp and sequence identity < 90% were predicted as insert sequences. The predicted insert sequences were further searched using BlastX analysis in MvirDB database (MvirDB; last accessed April 21, 2012; Zhou et al., 2007) to investigate the possible mobile genetic elements (e.g., conjugate transposons, transposases, and integrases) (E value ≤ 1e-7). Prophages or degraded prophages were predicted using PHAge Search Tool (PHAST), which is a web server that available at (http://phast.wishartlab.com) (Zhou et al., 2011).

### Data access

The raw reads have been deposited in the Sequence Read Archive under the following accession numbers: Pi-disease SRR5830884; Pi-health SRR5830885; Pn-disease SRR5830886; Pn-health SRR5830889.

## Author contributions

Experimental design: YFZ and JW; Clinical examination and sampling: MZ and YLZ; Experiments: YFZ, MZ, and YS; data analysis: YFZ, JW, YS, YLZ, and QZ; Manuscript revised: YFZ, JW, and MZ. All authors read and approved the final manuscript.

### Conflict of interest statement

The authors declare that the research was conducted in the absence of any commercial or financial relationships that could be construed as a potential conflict of interest.
