# Supplementary material for: Population-Genomic Insights into Variation in Prevotella intermedia and Prevotella nigrescens Isolates and Its Association with Periodontal Disease
Source: Front Cell Infect Microbiol. 2017 Sep 21;7:409. doi: 10.3389/fcimb.2017.00409 (PMC5613308; doi:10.3389/fcimb.2017.00409)
Supplement: Supplementary file 1 [file Table1.DOCX]

**Table S1 General information of 48 Prevotella isolates**

| **Group** | **Isolate name** | **Origin^**^** | **Clinical parameters of sampling site^##^** | | | |
| --- | --- | --- | --- | --- | --- | --- |
|  |  |  | **PD (mm)** | **AL (mm)** | **BOP** | **TM** |
| Pi-disease | Pi 47^*^-2^#^ | WYF, F, 65 | 8 | 4 | 3 | Ⅰ |
| Pi-disease | Pi 63-7 | MS, F, 32 | 7 | 5 | 4 | 0 |
| Pi-disease | Pi 64-10 | MS,F,32 | 6 | 4 | 4 | 0 |
| Pi-disease | Pi 94-5 | GCT, F, 24 | 6 | 3 | 4 | Ⅰ |
| Pi-disease | Pi 99-5 | JZF, F,34 | 5 | 3 | 4 | 0 |
| Pi-disease | Pi 101-1 | QJP, M, 35 | 5 | 2 | 4 | 0 |
| Pi-disease | Pi 106-8 | QQS,M, 42 | 5 | 2 | 4 | 0 |
| Pi-disease | Pi 107-3 | WZ, M, 40 | 5 | 2 | 4 | 0 |
| Pi-disease | Pi 116-1 | LBP, M, 29 | 5 | 1 | 3 | 0 |
| Pi-disease | Pi 119-3 | MH, M, 26 | 7 | 4 | 4 | Ⅰ− |
| Pi-disease | Pi 127-3 | GHF,M, 49 | 6 | 3 | 4 | 0 |
| Pi-disease | Pi 137-4 | FCQ, M, 47 | 9 | 5 | 4 | Ⅱ |
| Pi-disease | Pi 140-2 | ZQJ, M, 43 | 5 | 2 | 2 | 0 |
| Pi-disease | Pi 143-2 | ZHH, F, 24 | 5 | 2 | 3 | 0 |
| Pi-healthy | Pi 93-4 | GCT, F, 24 | 4 | 0 | 2 | 0 |
| Pi-healthy | Pi 102-8 | QJP, M, 35 | 4 | 0 | 0 | 0 |
| Pi-healthy | Pi 108-2 | WZ, M, 40 | 3 | 0 | 1 | 0 |
| Pi-healthy | Pi 114-1 | LW, M, 30 | 3 | 0 | 1 | 0 |
| Pi-healthy | Pi 118-6 | ZLP, F, 27 | 3 | 0 | 1 | 0 |
| Pi-healthy | Pi 139-3 | ZQJ, M, 43 | 2 | 0 | 1 | 0 |
| Pn-disease | Pn 11-1 | FYZ, M, 61 | 5 | 4 | 4 | 0 |
| Pn-disease | Pn 19-1 | SY, F, 25 | 6 | 3 | 4 | 0 |
| Pn-disease | Pn 20-4 | SY, F, 25 | 5 | 2 | 4 | 0 |
| Pn-disease | Pn 23-4 | CJY, M, 38 | 8 | 6 | 4 | Ⅰ− |
| Pn-disease | Pn 52-2 | WH, F, 23 | 6 | 2 | 4 | 0 |
| Pn-disease | Pn 98-1 | CY, F, 51 | 7 | 4 | 4 | 0 |
| Pn-disease | Pn 99-7 | JZF, F,34 | 5 | 3 | 4 | 0 |
| Pn-disease | Pn 104-1 | GDJ, F, 46 | 5 | 2 | 4 | 0 |
| Pn-disease | Pn 106-11 | QQS, M, 42 | 5 | 2 | 4 | 0 |
| Pn-disease | Pn 112-7 | LY, F, 28 | 5 | 1 | 1 | 0 |
| Pn-disease | Pn 116-4 | LBP, M, 29 | 5 | 1 | 3 | 0 |
| Pn-disease | Pn 122-3 | PFJ, F, 44 | 6 | 2 | 3 | 0 |
| Pn-disease | Pn 129-1 | YGC, M, 30 | 5 | 2 | 3 | 0 |
| Pn-disease | Pn 131-8 | ZW, M, 22 | 6 | 2 | 2 | 0 |
| Pn-disease | Pn 133-8 | YX, F, 32 | 6 | 3 | 2 | 0 |
| Pn-disease | Pn 135-8 | WY, F, 26 | 5 | 3 | 3 | 0 |
| Pn-disease | Pn 152-4 | WSS, F, 22 | 5 | 2 | 3 | 0 |
| Pn-healthy | Pn 77 | WYY, F, 20 | 4 | 0 | 4 | 0 |
| Pn-healthy | Pn 78-1 | WYY, F, 20 | 3 | 0 | 2 | 0 |
| Pn-healthy | Pn 84-3 | LGZ, F, 60 | 4 | 0 | 2 | 0 |
| Pn-healthy | Pn 85-1 | ZLL, F, 26 | 3 | 0 | 2 | 0 |
| Pn-healthy | Pn 102-1 | QJP, M, 35 | 4 | 0 | 0 | 0 |
| Pn-healthy | Pn 114-8 | LW, M, 30 | 3 | 0 | 1 | 0 |
| Pn-healthy | Pn 120 | MH, M, 26 | 4 | 0 | 3 | 0 |
| Pn-healthy | Pn 123-2 | ZYQ, F, 60 | 3 | 0 | 0 | 0 |
| Pn-healthy | Pn 128-7 | GHF, M, 49 | 3 | 0 | 2 | 0 |
| Pn-healthy | Pn 136-3 | WY, F, 26 | 2 | 0 | 2 | 0 |
| Pn-healthy | Pn 149 | YYX, F, 37 | 3 | 0 | 2 | 0 |

^*^The number of sampling sites, the same number means from the same site

^#^ The number of isolates, e.g. Pi 47-2 means number 2 *P. intermedia* isolate from number 47 sampling site.

^**^Origin: basic information of subjects who provided the isolates, including the patient name code, gender (M: male; F: female) and age.

^##^ Clinical parameters of sampling site: PD: Probing depth; AL: Attachment Loss; BOP: Bleeding on probing; TM: Tooth Mobility.
