# Supplementary material for: Population-Genomic Insights into Variation in Prevotella intermedia and Prevotella nigrescens Isolates and Its Association with Periodontal Disease
Source: Front Cell Infect Microbiol. 2017 Sep 21;7:409. doi: 10.3389/fcimb.2017.00409 (PMC5613308; doi:10.3389/fcimb.2017.00409)
Supplement: Supplementary file 10 [file DataSheet1.pdf]

Supplementary Material

Population-genomic insights into variation in *Prevotella intermedia* and *Prevotella nigrescens* isolates and its association with periodontal disease

Yifei Zhang[#*], Min Zhen[#], Yalin Zhan, Yeqing Song, Qian Zhang, Jinfeng Wang[*]

Correspondence:
[*]Jinfeng Wang
wangjf@biols.ac.cn

[*]Yifei Zhang
wingsflying2000@163.com

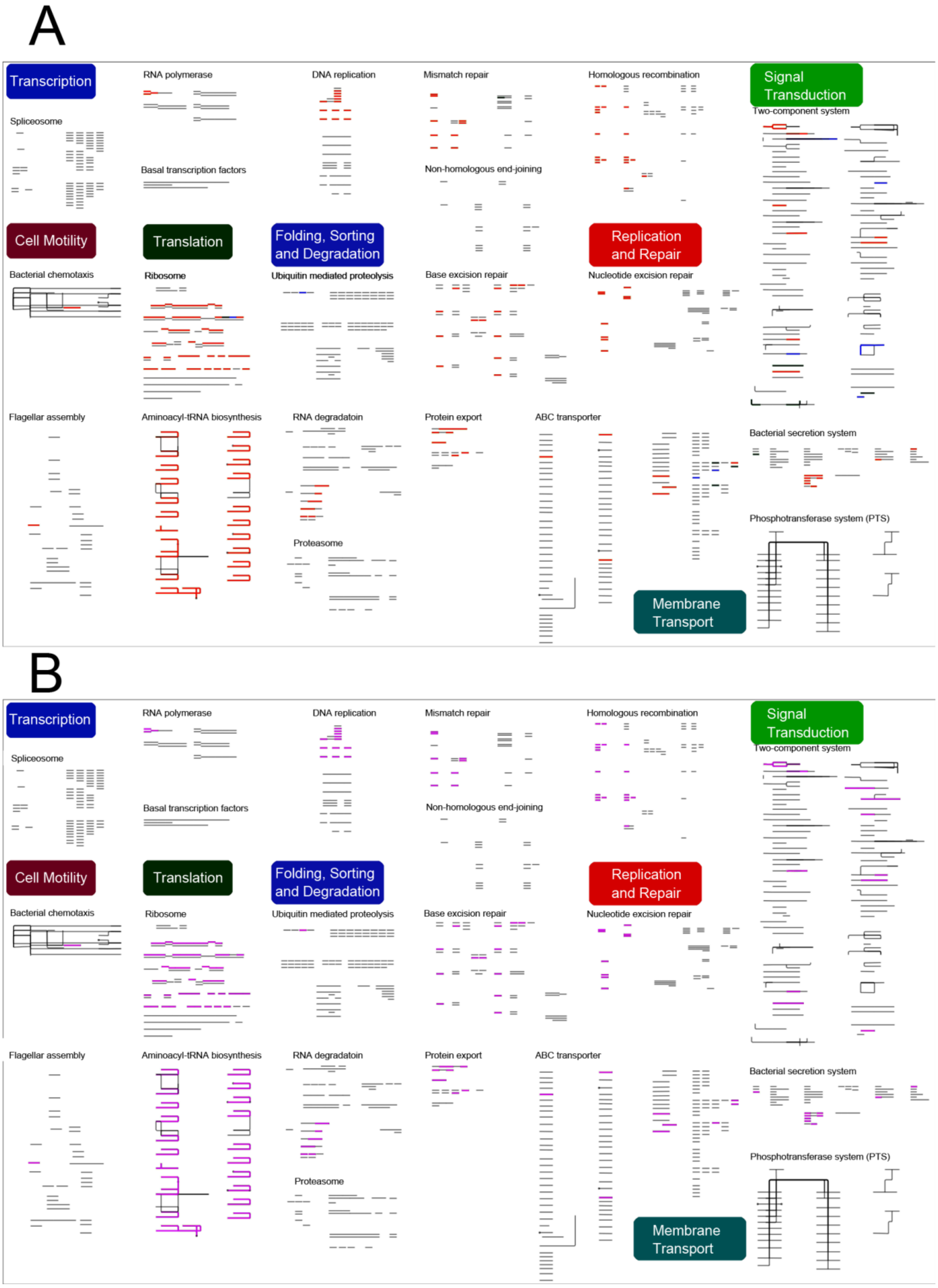

Supplementary Figure 1. A. Regulatory pathway reconstructions of Pi-disease and Pn-disease. Pipelines colored in orange represent the common pathways shared by both samples. The pipelines colored in purple and green represent the specific pathway in Pi-disease and Pn-disease, respectively; B. Regulatory pathway reconstructions of Pi-health and Pn-health. Pipelines colored in pink represent the common pathways shared by both samples; no specific pathway was seen in Pi-health or Pn-health.

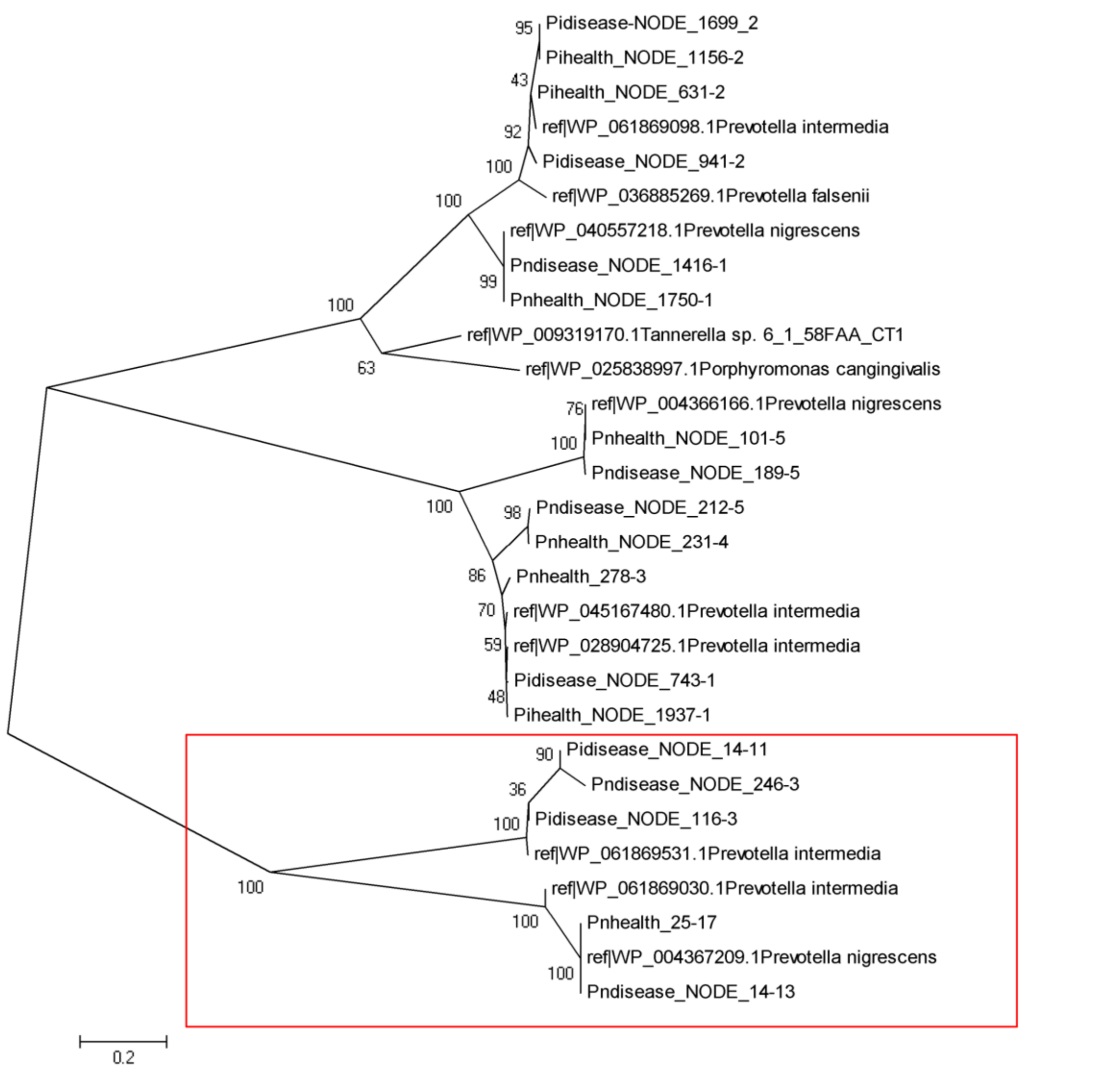

Supplementary Figure 2. A phylogenetic tree of LPS biosynthesis proteins in type culture strains from NCBI database and in clinical strains from Pi-disease, Pi-health, Pn-disease and Pn-health groups. The rectangle highlights that four disease-derived isolates clustered within a highly distinct and uniform clade.

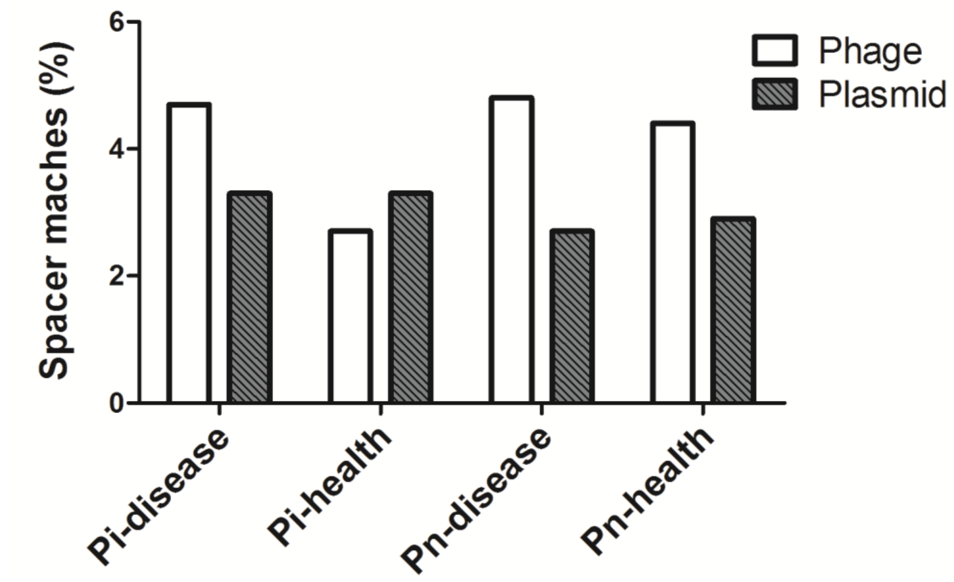

Supplementary Figure 3. Proportion of the CRISPR spacers that homology to phage (light bars) and plasmid (dark bars) array sequences in each sample group.
