# Supplementary figures and images for: Population-Genomic Insights into Variation in Prevotella intermedia and Prevotella nigrescens Isolates and Its Association with Periodontal Disease
Source: Front Cell Infect Microbiol. 2017 Sep 21;7:409. doi: 10.3389/fcimb.2017.00409 (PMC5613308; doi:10.3389/fcimb.2017.00409)

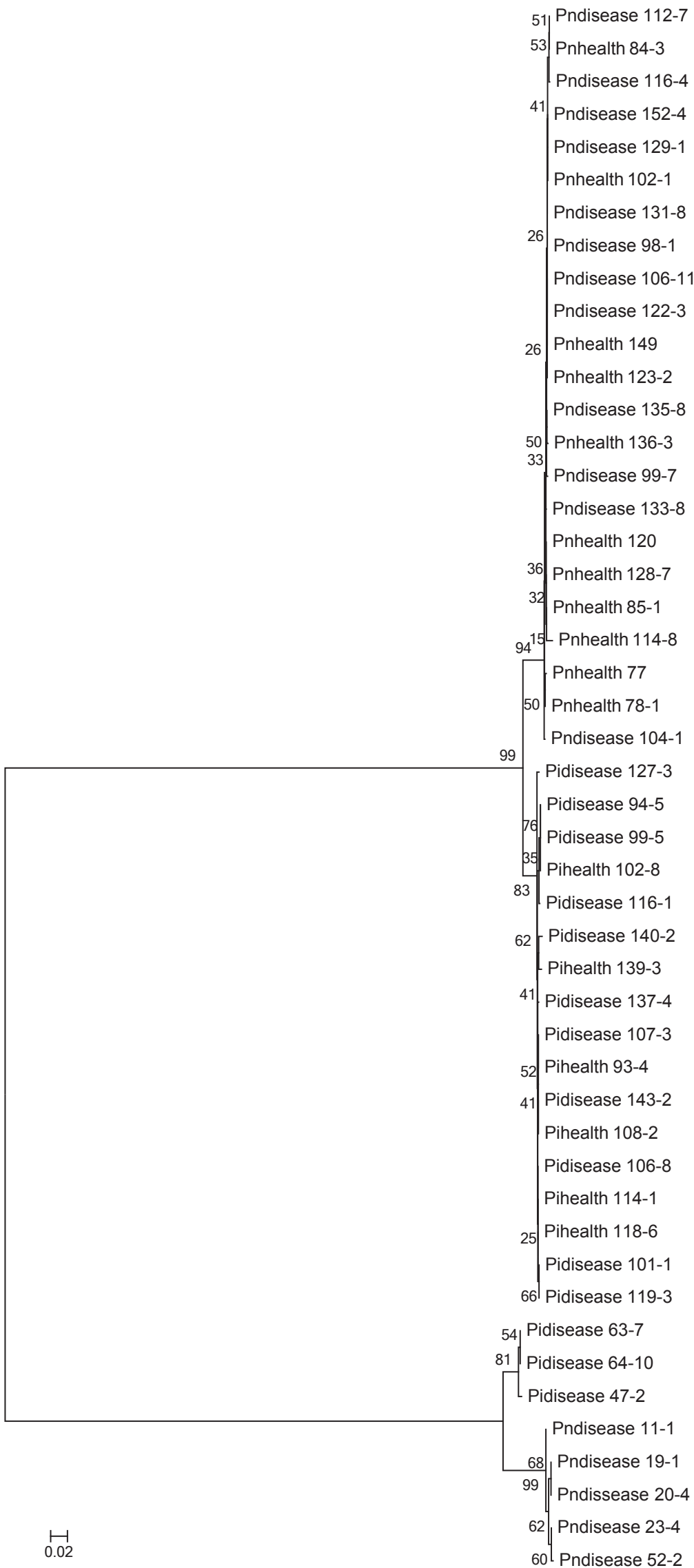

Supplement: Supplementary file 11 [file DataSheet2.pdf]
